# Knock-Down of IL-1Ra in Obese Mice Decreases Liver Inflammation and Improves Insulin Sensitivity

**DOI:** 10.1371/journal.pone.0107487

**Published:** 2014-09-22

**Authors:** Niclas Franck, Michael Maris, Sarah Nalbandian, Saswata Talukdar, Simon Schenk, Hans-Peter Hofmann, David Bullough, Olivia Osborn

**Affiliations:** 1 Division of Endocrinology and Metabolism, Department of Medicine, University of California San Diego, La Jolla, California, United States of America; 2 Clinical and Experimental Endocrinology, Catholic University Leuven, Leuven, Belgium; 3 Pfizer Inc. Research Laboratories, Cambridge, Massachusetts, United States of America; 4 Department of Orthopedic Surgery, University of California San Diego, La Jolla, California, United States of America; Institut d'Investigacions Biomèdiques August Pi i Sunyer, Spain

## Abstract

Interleukin 1 Receptor antagonist (IL-1Ra) is highly elevated in obesity and is widely recognized as an anti-inflammatory cytokine. While the anti-inflammatory role of IL-1Ra in the pancreas is well established, the role of IL-1Ra in other insulin target tissues and the contribution of systemic IL-1Ra levels to the development of insulin resistance remains to be defined. Using antisense knock down of IL-1Ra in vivo, we show that normalization of IL-1Ra improved insulin sensitivity due to decreased inflammation in the liver and improved hepatic insulin sensitivity and these effects were independent of changes in body weight. A similar effect was observed in IL1-R1 KO mice, suggesting that at high concentrations of IL-1Ra typically observed in obesity, IL-1Ra can contribute to the development of insulin resistance in a mechanism independent of IL-1Ra binding to IL-1R1. These results demonstrate that normalization of plasma IL-1Ra concentration improves insulin sensitivity in diet- induced obese mice.

## Introduction

Insulin resistance is a pathophysiological defect commonly found in obese individuals, and is an important predictor for the progression to type 2 diabetes [1]. The incidence of obesity and associated insulin resistance has risen dramatically in the past 20 years and so understanding the pathways driving the development of insulin resistance is of high importance. Tissue inflammation is now recognized as a major cause of impaired insulin sensitivity in obesity [2], [3] and has been observed in all classical insulin target tissues including fat, liver and muscle. Interleukin 1 Beta (IL-1β) is an important proinflammatory cytokine that binds to the type 1 IL-1 Receptor (IL-1R1) and has well described proinflammatory effects [4]. Signal transduction is elicited by interaction of the IL-1R1 with an accessory protein (IL-1RAcP) [5], [6]. The endogenous antagonist IL-1R Antagonist (IL-1Ra), also binds to the IL-1R1 but does not initiate signal transduction [7]. IL-1Ra is classically viewed as an anti-inflammatory cytokine that acts as a selective, competitive receptor antagonist at the IL-1R1 by blocking the actions of IL-1β [8] and the balance in expression between IL-1β and IL-1Ra is important in many inflammatory diseases [9].

While the anti-inflammatory role of IL-1Ra in the pancreas is well established in both mice [4], [10], [11] and humans [12], [13], the role of IL-1Ra in other insulin target tissues and the general role of systemic levels of IL-1Ra in the development of obesity and insulin resistance is still unclear. A number of studies over the last decade have raised the question of whether the high levels of IL-1Ra observed in obesity may contribute to the development of insulin resistance. In obese human patients, circulating levels of IL-1Ra are ∼6.5 times higher than lean subjects and, interestingly, the levels of IL-1Ra in plasma correlate more closely with insulin resistance than BMI [14], suggesting an important link between IL-1Ra and insulin resistance. Indeed, elevated systemic concentrations of IL-1Ra are associated with an increased risk of developing type 2 diabetes [15], [16], and in retrospective studies this increase in IL-1Ra accelerated prior to type 2 diabetes diagnosis [17]. In a recent study IL-1Ra was even identified as a novel biomarker for clinically incident diabetes, over and above the classical risk factors such as BMI and waist: hip ratio [18]. Supporting a possible role in the development of insulin resistance, thiazolidinedione (TZD) treatment significantly reduced levels of IL-1Ra in patients with metabolic syndrome [19], as well as in cell culture studies where TZDs inhibited the production of IL-1Ra from proinflammatory adipocytes [20].

Furthermore, studies in rodents have further confirmed the link between IL-1Ra and insulin resistance. In a short five day study, administration of IL-1Ra led to a decrease in whole body insulin sensitivity in rats [21] while whole body deletion of IL-1Ra significantly improved insulin sensitivity in mice [21]–[23]. However, the IL-1Ra whole body KO mouse is particularly lean (<20g by 16wks age) which raises the possibility that the complete absence of IL-1Ra from birth may cause growth or sickness issues [23]. Taken together, these results provide a strong link between IL-1Ra and insulin action but to better define the role of IL-1Ra in vivo, in the present study we investigated the hypothesis that normalization of IL-1Ra would improve insulin sensitivity. To address this hypothesis we used antisense oligonucleotides (ASO) to knock down both the secreted and intracellular forms of IL-1Ra in high fat diet (HFD)-fed obese mice. Overall our studies show that reduction of IL-1Ra levels (to approximately half the normal circulating levels in obese mice) improves hepatic insulin sensitivity.

## Materials and Methods

### Chemicals and Reagents

All chemicals were purchased from Sigma (USA) unless otherwise noted.

### In vitro knock-down of IL-1Ra

Antisense oligonucleotides (ASO) against IL-1Ra were a gift from Pfizer Inc. and were synthesized as described by Stanton, 2012 [24]. IL-1Ra ASO-1: 5′ - ßA*ßT*mC*dA*dG*dG*dC*dA*dG*dT*dT*mG*ßG*ßt - 3′; IL-1Ra ASO-2: 5′-ßT*ßT*dG*dG*dT*dC*dT*dG*dG*dA*dC*dT*ßG*ßT - 3′; Control ASO: 5′-βZ*ßG*dT*dC*dT*dA*dT*dG*dT*dA*ßT*ßA*ßG - 3′ (*, phosphorothioate backbone link; LNA, locked nucleic acid; Z, 5-methyl cytosine; ß, LNA base; m, 2′-OMethyl nucleoside; A, adenosine monomer; C, cytidine monomer; G, guanosine monomer; T, thymidine monomer).

The efficiency of knock-down of IL-1Ra expression was tested in Hepa 1–6 mouse hepatoma cells by gymnotic delivery. In brief, cells were seeded in 96-well plates at 2,500 cells per well and treated with IL-1Ra ASOs without transfection reagent at varying concentrations. Control cells were incubated with medium only. After 5 days cells were lysed and IL-1Ra mRNA knock-down was analyzed by quantitative PCR.

### Mice

All experiments were approved by and conducted in accordance with the Animal Care Program at the University of California, San Diego. All surgery was performed under ketamine anesthesia, and every effort was made to minimize suffering. Male C57BL/6 and IL-1R1 KO were purchased from Jackson Laboratories and at 8 weeks of age. Mice were fed HFD (D12492, Research Diets, 60% of kcal derived from fat) *ad libitum* for 12 weeks to generate diet induced obese (DIO) mice. For acute studies, DIO mice were injected (subcutaneously, 10 mg/kg) once with IL-1Ra ASO (1 or 2) or control ASO and sacrificed one week later. For chronic studies, DIO mice at 20 weeks of age mice were divided into two groups, IL-1Ra ASO-1 or control ASO and both groups were treated twice a week by subcutaneous injection at a dose of 10 mg/kg for 6 weeks while maintained on HFD. At the end of the study (26 wks of age) all the mice were sacrificed.

### Metabolic studies

For Glucose tolerance tests (GTT), an intraperitoneal (IP) injection of glucose (1 g/kg) was administered in mice fasted for 6 hours. Blood was collected for measurement of plasma insulin concentration before the injection and 10 and 90 minutes after glucose injection. Blood glucose concentration was assessed at indicated time points. For insulin tolerance tests (ITT), an IP dose of 0.35 U/kg insulin was administered, and blood glucose was measured at the indicated time points. Hyperinsulinemic euglycemic clamp studies were conducted as previously described [25]. Briefly, dual catheters (MRE-025, Braintree Scientific) were implanted in the right jugular vein and mice were allowed to recover for 3 days before the clamp procedure. After 6 hr fasting, the clamp experiments began with a constant infusion (5 µCi/hr) of D-[3-^3^H] glucose (Du Pont-NEN, Boston, MA). After 90 minutes of tracer equilibration and basal sampling at −10 and 0 minutes, glucose (50% dextrose, variable infusion, Abbott) and tracer (µCi/hr) plus insulin (8 mU/kg/min) was infused via the jugular vein cannulae. Blood was taken from tail clips at 10 minute intervals and analyzed for glucose. Steady-state conditions (120 mg/dl ±5 mg/dl) were confirmed at the end of the clamp by ensuring that glucose infusion and plasma glucose levels were maintained constant for a minimum of 30 min. Blood samples were taken at −10, 0 (basal), 110 and 120 (end of experiment) minutes to determine glucose-specific activity and insulin and FFA levels. Hepatic glucose production (HGP) and glucose disposal rate (GDR) were calculated in the basal state and during the steady-state portion of the clamp. Tracer-determined rates were quantified by using the Steele equation [26]. At steady state, the rate of glucose disappearance, or total GDR, is equal to the sum of the rate of endogenous HGP plus the exogenous (cold) GIR. The IS-GDR is equal to the total GDR minus the basal glucose turnover rate. Glucose was measured using a blood glucose monitor and test strips (Easy Step, Deerfield Beach, FL, USA).

### Indirect calorimetry and measurement of core body temperature and activity

After 4 weeks of treatment mice were placed into Comprehensive Lab Animal Monitoring System (CLAMS, Columbus Instruments) metabolic cages. Data were recorded after 48 hours of acclimatization to the chambers under ambient room temperature maintained at 25°C, beginning from the onset of the light cycle 24 hr for three days. Measurement of CBT and locomotor activity was performed as previously described [27]. In brief, radiotelemetry devices (Mini Mitter, Respironics) were surgically implanted into the intraperitoneal cavity and core body temperature and activity was recorded every 15 minutes over a 24 hr period.

### RNA Isolation and Quantitative-PCR

Total RNA was isolated using Trizol as described by the manufacturer. (Trizol, Invitrogen, CA). First strand cDNA was synthesized using a High-Capacity cDNA Reverse Transcription Kit (Applied Biosystems, Foster City, CA). For quantitative PCR (qPCR) the samples were run in 20 µl reactions (iTaq SYBRgreen supermix, Biorad) using a stepOnePlus Real Time PCR system, Applied Biosystems). Gene expression levels were calculated after normalization to the standard housekeeping gene *ActB* using the ΔΔ*C*_T_ method as described previously [28], and results are expressed relative to the control group mean value. Primer sequences are described in table S1).

### Measurement of serum parameters

IL-1Ra serum levels were measured using a Mouse IL-1ra/IL-1F3 Quantikine ELISA Kit (R&D Systems, Minneapolis, MN, USA). Plasma concentrations of Interleukin 10 (IL-10), Interleukin 1 beta (IL-1β), Interleukin 6 (IL-6), Monocyte chemotactic protein 1 (MCP-1) and Tumor necrosis factor alpha (TNF-mor necrosis factorleukin 10 (IL-10), Interleukin 1 beta (ILBillerica, MA, USA). Plasma insulin levels were measured by ELISA (ALPCO diagnostics, USA). Plasma free fatty acid (FFA) levels were measured enzymatically using a commercially available kit (NEFA C; Wako Chemicals USA). Triglyceride (TG) levels were measured enzymatically using a commercially available kit (L-Type TG M, Wako Chemicals, USA). Liver enzymes alanine aminotransferase (ALT), aspartate aminotransferase (AST) and glutamate dehydrogenase (GDH) were measured using assay kits (Sigma, MO, USA).

### Statistical analyses

Statistical analyses were performed using the Student's *t*-test to compare two groups. One –way ANOVA followed by a Tukey post hoc test was used to assess significance between three groups. Two-way anova with repeated measures followed by the Holm–Sidak test was used to compare two or more groups at different time points. Significance was defined as p value equal or less than less than 0.05 (*). The results are shown as means ± SEM. Graph Pad Prism (Graph Pad, San Diego, CA) was used for all statistical analyses.

## Results

### IL-1Ra ASO reduces expression of IL-1Ra in vitro and acutely in vivo

**Figure 1:**
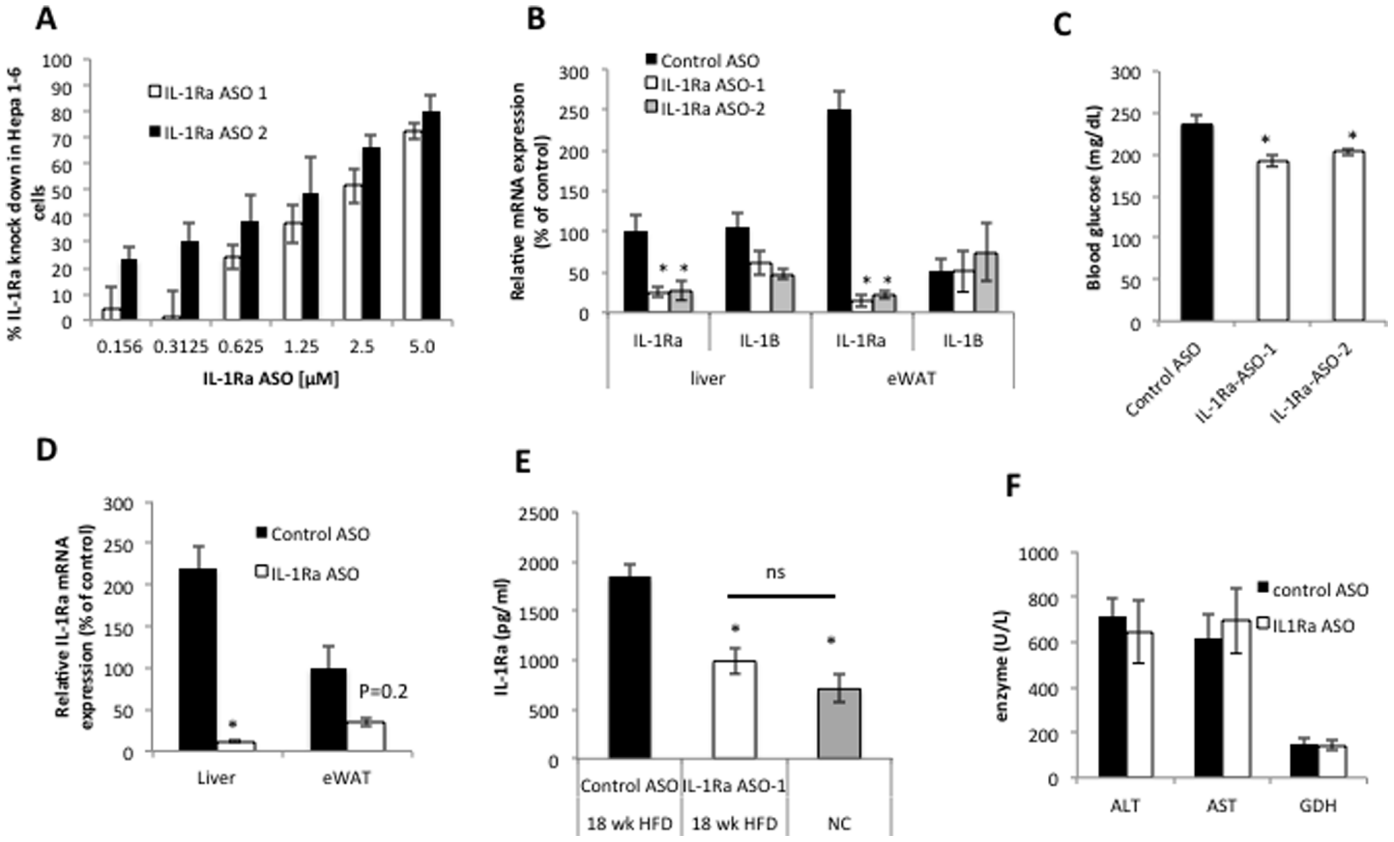
Antisense oligonucleotide (ASO) mediated knock-down of IL-1Ra transcript in vitro and in vivo using locked nucleic acids (LNAs). A) QPCR analysis of IL-1Ra mRNA expression after ASO treatment in Hepa 1–6 cells, each concentration was run in duplicate. B) QPCR analysis of IL-1Ra and IL-1β mRNA levels in liver and epididymal adipose (eWAT) from mice one week after IL-1Ra ASO administration. C) Blood glucose concentration in mice one week after IL-1Ra ASO treatment. D. QPCR analysis of IL-1Ra mRNA expression in liver and eWAT after 6 weeks of treatment of IL-1Ra ASO or control ASO. E. Plasma IL-1Ra concentration in obese mice treated with IL-1Ra ASO or control ASO for 6 weeks compared with aged matched lean control mice fed normal chow (NC). F. Analysis of liver enzyme levels after IL-1Ra ASO treatment in HFD-fed mice (ALT, alanine aminotransferase; AST, aspartate aminotransferase; GDH, glutamate dehydrogenase). *P<0.05 compared with control treated mice, QPCR results are normalized to beta actin mRNA and expressed relative to control treatment. Differences between groups were analyzed by one way ANOVA followed by Tukey post hoc tests, n = 5–8 mice per group, * p<0.05 compared to HFD-fed control treated group; ns, not significant.

In vitro studies in Hepa 1–6 cells using two different ASOs (IL-1Ra ASO-1 and IL-1Ra ASO-2) directed against IL-1Ra revealed a dose dependent knock down of IL-1Ra mRNA(Fig. 1*A*). To determine whether antisense treatment reduced the expression of IL-1Ra in vivo we administered the IL-1Ra ASOs and control ASO by subcutaneous injection, dose 10 mg/kg, to obese mice (fed a 60% HFD for 12 weeks). One week after ASO injection mice were sacrificed IL-1Ra mRNA expression was analyzed by QPCR. Both IL-1Ra ASOs resulted in significant reduction of IL-1Ra in both liver and epididymal adipose tissue (Fig. 1*B*) while closely related family member IL-1β expression was not significantly changed in either tissue (Fig 1B). Furthermore, after 1 week of treatment, both IL-1Ra ASOs resulted in a significant decrease in blood glucose compared with control ASO treatment (Fig 1C), suggesting these ASOs act specifically on IL-1Ra and the glucose lowering effects are less likely to be attributable to off-target secondary effects. IL-1Ra ASO-1 at a dose of 10 mg/kg was selected for use in the chronic studies.

### Chronic treatment with IL-1Ra ASO reduces expression of IL-1Ra in vivo

To determine the extent of IL-1Ra knock down in HFD fed mice after chronic treatment (6weeks) with IL-1Ra ASO or control ASO in vivo we measured IL-1Ra mRNA expression levels in tissues as well as circulating protein levels of IL-1Ra. After 6 weeks of treatment the expression of IL-1Ra mRNA was decreased by ∼97% (p<0.05) in the liver and 63% (p = 0.2) in epididymal fat (Fig. 1D) but was not changed in many other tissues (figure S1A). Furthermore, circulating levels of IL-1Ra were reduced by approximately 50% by IL-1Ra ASO treatment compared with control ASO treatment, to similar levels found in normal chow fed, lean mice (Fig. 1E). Circulating levels of other inflammatory mediators including MCP-1, TNF-α and IL-6 were not different between groups (figure S1C), suggesting IL-1Ra ASO treatment did not activate the innate immune system. Importantly, ASO treatment did not cause liver toxicity, as no difference was observed in the circulating levels of liver enzymes alanine aminotransferase (ALT), aspartate aminotransferase (AST) and glutamate dehydrogenase (GDH) after 6 weeks of treatment in HFD-fed obese mice (Fig 1F).

### Effect of IL-1Ra knock-down on body weight and insulin resistance in obese-insulin resistant mice

**Figure 2:**
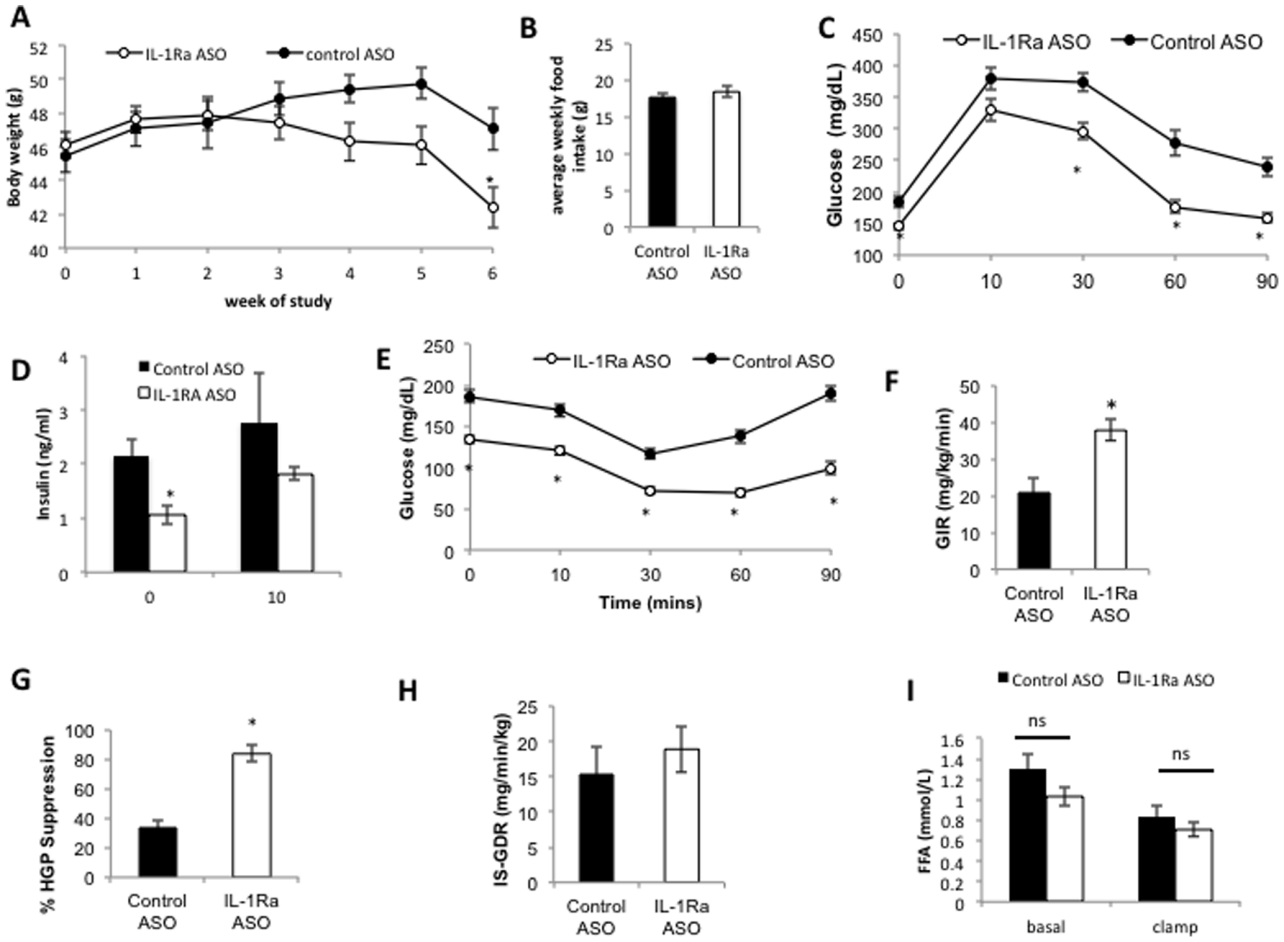
IL-1Ra ASO treatment in HFD-fed obese mice results in decreased body weight (A) despite similar food intake (B) (food intake measured weekly and presented as average food intake over first of 4 weeks of study), improved glucose tolerance (GTT after 3 wks of treatment, treatment p<0.0001, time, p<0.0001, interaction, p = 0.0285) (C) with lower fasting insulin levels (D) and improved insulin sensitivity (ITT at 4 wks of treatment, treatment p<0.001, time p<0.0001, interaction p<0.0001) (E). n = 12 mice per group, *P<0.05 compared with control treated mice. (F–I) Hyperinsulinemic/euglycemic clamp studies in IL-1Ra ASO treated and control treated HFD mice. F) Glucose infusion rate (GIR), G)% suppression of hepatic glucose production (HGP), H) Insulin stimulated glucose disposal rate (IS-GDR), I) Free fatty acids (FFA). A repeated measures 2-way ANOVA showed a significant effect of treatment, time and interaction in both GTT and ITT experiments. A students t-test was used to asses significance between groups (GIR,% suppression and IS-GDR), a 2-way ANOVA with post hoc Sidak test showed no significant effect on FFA concentration between treatment groups, but a significant effect within groups between basal and clamped samples. n = 5−8 mice per group, * p<0.05 compared to HFD-fed control treated group; ns, not significant.

In vivo, IL-1Ra ASO treatment of HFD-fed obese mice resulted in weight loss from approximately 4 weeks of treatment and after 6 weeks of treatment IL-1Ra ASO treated mice were significantly lighter than control ASO treated (Fig. 2*A*), despite no significant difference in food intake (Fig. 2*B*). IL-1Ra ASO treatment resulted in significant improvements in glucose tolerance (Fig. 2*C*, figure *S2B*), largely due to a decrease in basal glucose, as when calculated as percentage change in glucose from initial levels the effect is less pronounced (figure S2A), IL-1Ra ASO treatment reduced fasting insulin (Fig. 2*D*), and improved insulin sensitivity as assessed by an insulin tolerance test (Fig. 2*E* and figure S2C, D).

Despite the differences observed in body weight in the whole cohort we were able to select a subgroup of body weight matched mice (average BW 44 g) from the lighter control ASO treated mice and heavier IL-1Ra ASO-treated mice to study body weight independent effects on insulin sensitivity. We performed hyperinsulinemic- euglycemic clamp studies in this body weight matched, HFD-fed, obese cohort of mice (n = 6−7 per group) (Fig. 2*F*–*I*) to determine which insulin target tissue was responsible for the systemic improvements in insulin sensitivity. These clamp experiments confirmed that the IL-1Ra ASO treated mice have improved systemic insulin sensitivity (Fig. 2*F*) that was the result of improved insulin-mediated suppression of hepatic glucose production (Fig. 2*G*). No significant difference in insulin sensitivity was observed in muscle (as measured by the insulin-stimulated glucose disposal rate; Fig. 2*H*) or adipose tissue (as measured by suppression of plasma FFA concentration; Fig. 2*I*). These studies demonstrate that IL-1Ra expression levels play an important role in modulating insulin sensitivity. These clamp results show that, reduced expression of IL-1Ra had beneficial effects to improve hepatic insulin sensitivity as well as systemic insulin sensitivity.

### Effect of IL-1Ra ASO treatment in the liver of obese-insulin resistant mice

**Figure 3:**
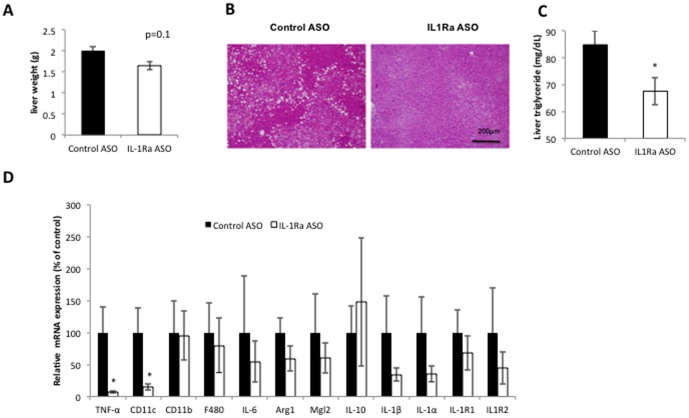
IL1Ra ASO treatment reduces liver triglyceride and improved liver inflammation. A) Liver weight (g), B) H&E stain of liver. C) Liver triglyceride content after 6 weeks of IL-1Ra ASO or control ASO treatment in HFD fed mice. D) QPCR analysis of inflammatory gene expression in the liver of obese mice treated with IL-1Ra ASO or control ASO, results are normalized to beta actin mRNA and expressed relative to control ASO treatment. * p<0.05 compared to HFD-fed control treated group, n = 5−8 mice per group.

IL-1Ra ASO treated mice had slightly lighter liver weights (p = 0.1), significantly reduced triglyceride levels (Fig. 3*B,C*). Expression of proinflammatory cytokine tumor necrosis factor alpha (TNF-α) and proinflammatory macrophage marker (CD11c) genes were also significantly reduced (Fig. 3*D*), indicating that IL-1Ra ASO treatment results in decreased liver inflammation in HFD- fed mice. These studies were conducted in weight matched obese mice and so IL-1Ra ASO mediated improvement in liver triglyceride levels and reduced inflammation are independent of changes in body weight. Analysis of expression of hepatic gluconeogenic genes after IL-1Ra ASO treatment revealed a slight reduction in PEPCK mRNA but no change in G6Pase (figure S1B).

### Effect of IL-1Ra ASO treatment on energy expenditure in obese-insulin resistant mice

**Figure 4:**
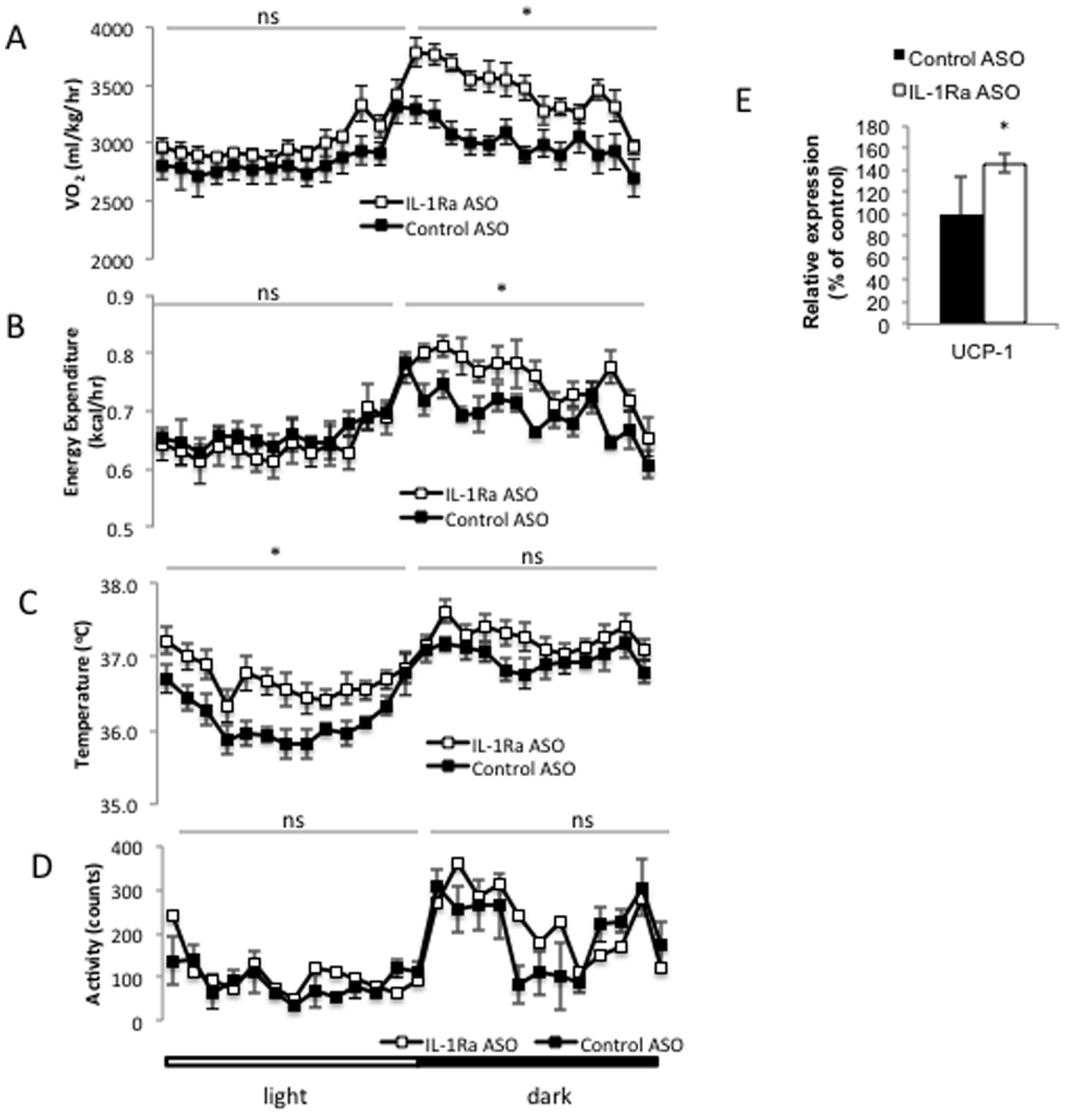
IL-1Ra ASO treatment results in increased energy expenditure. Oxygen consumption (A) and energy expenditure (B) were measured in CLAMS cages over a 48 hr period in week 4 of the study. core body temperature (C) in HFD-fed IL-1Ra ASO treated mice compared with control ASO treated mice. D) Activity is unchanged between groups. E) Quantitative PCR analysis of uncoupling protein 1 (UCP-1) in BAT. * p<0.05 compared to HFD-fed control treated group, n = 5 mice per group, ns, not significant.

The observation that IL-1Ra ASO treatment in obese mice resulted in a decrease in body weight (Fig. 2A) without associated differences in food intake (Fig. 2*B*) prompted us to measure the effects of treatment on energy expenditure. IL-1Ra ASO treatment for four weeks in obese mice resulted in an increase in oxygen consumption (Fig. 4*A*), and a significant increase in energy expenditure (Fig. 4*B*), specifically in the dark cycle. Evaluation of core body temperature (CBT) in the IL-1Ra ASO treated mice revealed an increase in CBT (Fig. 4*C*) that was not dependent on changes in locomotor activity (Fig. 4*D*). The increase in CBT independent of activity suggests that IL-1Ra ASO treatment may have additional effects driving increased thermogenesis. Indeed, quantitative PCR analysis revealed that transcript levels of *Ucp-1* are increased in the BAT of IL-1Ra ASO treated mice (Fig. 4E).

### Effect of IL-1Ra ASO treatment on adipose tissue

**Figure 5:**
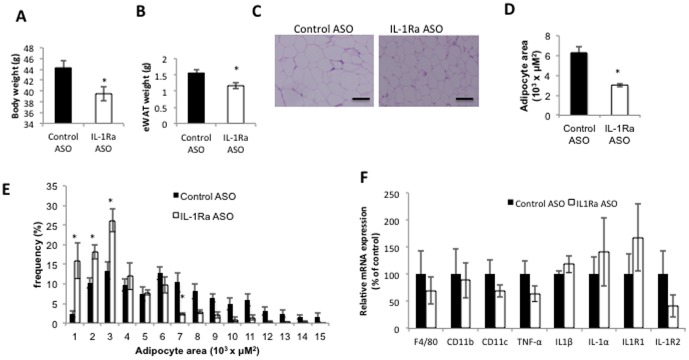
IL-1Ra ASO treatment effect on adipose tissue. After 6 weeks of treatment with IL-1Ra ASO mice have A) significantly reduced body weight, and B) reduced epididymal fat mass. C) H&E staining of visceral adipose tissue shows reduced adipocyte cell size. Scale bars  = 50 µm. Representative figures are presented from the analyses of five different mice per group. D) Quantification of average adipocyte cell area E) Histogram of adipocyte area in control treated mice (black bars), IL-1Ra ASO treated mice (white bars), using Image Gauge software, version 4 (FujiFilm). (n = 5 mice per group), F) QPCR analysis of inflammatory gene expression in adipose tissue. * p<0.05 compared to HFD-fed control treated group, n = 5−8 mice per group.

Chronic IL-1Ra ASO treatment (for 6 weeks) in obese mice results in decreased body weight (Fig. 5A), and decreased weight of the epididymal fat (eWAT) (Fig. 5B). We observed a significant decrease in adipocyte cell size (Fig. 5C,D,E) consistent with reduction in adipocyte cell size noted in whole body IL-1Ra KO mice [23]. Expression of proinflammatory markers in adipose tissue was not significantly different in IL-1Ra ASO compared with control ASO treated mice (Fig. 5F).

### Effects of IL-1Ra not mediated through Interleukin 1 Receptor 1 (IL-1R1)

**Figure 6:**
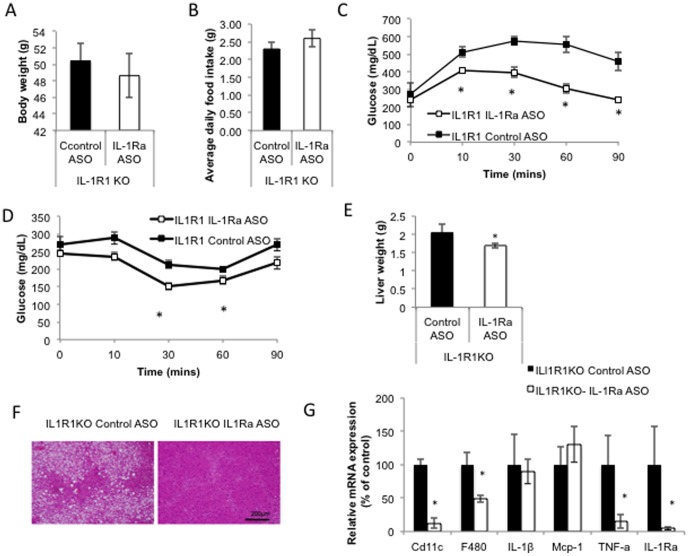
IL-1Ra ASO treatment improves glucose tolerance and insulin sensitivity independent of IL-1R1 signaling. A) Body weight, B) Average food intake, (measured weekly for 6 weeks). C. GTT after 5 weeks of treatment, (treatment p<0.035 time p<0.0001, interaction, 0.5554), D. ITT after 5.5 wks of treatment, (treatment p<0.0188, time p<0.0001, interaction p = 0.0058). E) Liver weight, F) H&E stain of liver, G) QPCR analysis of inflammatory gene expression, in IL-1R1KO mice fed with HFD for 12 weeks and then treated with IL-1Ra ASO or control ASO for 6 weeks, (n = 4 mice per group, * p<0.05 compared to HFD-fed control treated group). A repeated measures 2-way ANOVA was used to compare the effect of IL-1Ra ASO and Control ASO on GTT and ITT experiments.

To determine if the improvements in insulin sensitivity observed were through the actions of IL-1Ra signaling at IL-1R1 we treated HFD-fed IL-1R1 KO mice with IL-1Ra ASO. Body weight (Fig. 6*A*) and food intake (Fig. 6*B*) were not significantly different between IL-1R1 KO mice treated with IL-1Ra ASO or control ASO. We observed significant improvements in glucose tolerance (GTT, Fig. 6C) and insulin sensitivity (ITT, Fig. 6D) in the IL-1R1 KO treated with IL-1Ra ASO. However, when plotted as percentage change from basal (figure S2 E,G) or as area under the curve (figure S2 F,H) the effects of IL-1Ra ASO treatment in IL-1R1 KO mice were less pronounced. The liver of IL-1R1 KO mice treated with IL-1Ra ASO was significantly lighter than control treated IL-1R1 KO mice (Fig. 6E) and showed less steatosis (Fig. 6F) and significantly reduced inflammation (Fig. 6G). The improved glucose tolerance and reduced liver inflammation observed after IL-1Ra ASO treatment in IL-1R1 KO mice (similar to that observed in WT, diet induced obese mice treated with IL-1Ra ASO) confirm that these insulin sensitizing effects are independent of signaling at the IL-1R1 and occur through an alternate mechanism.

## Discussion

IL-1Ra is up-regulated during diet-induced obesity [21] and both adipose tissue and the liver are major sources of IL-1Ra [20], [29]. Previous studies have reported that IL-1Ra may play a role in the development of insulin resistance [14]–[20]. To determine whether decreasing systemic IL-1Ra levels in obesity has therapeutic potential, we treated HFD-fed obese mice with antisense oligonucleotides (ASOs) resulting in 50% reduction in the systemic concentration of IL-1Ra, to similar levels measured in age matched, lean normal chow fed mice. The extent of reduction in expression levels is comparable with may other published reports on the use of locked nucleic acid ASOs to inhibit gene expression [30]–[33]. We report for the first time that normalization of IL-1Ra by antisense-mediated knock-down of IL-1Ra expression improves hepatic insulin sensitivity and decreases body weight in diet induced obese mice. Importantly, studies in IL-1R1 KO mice revealed that the IL-1Ra ASO mediated improvement in insulin sensitivity was independent of IL-1R1.

Differences in body weight and the degree of obesity strongly impact insulin action. An important finding was that in HFD-fed mice, IL-1Ra ASO treatment not only improved insulin action, but also reduced body weight by ∼10% after 6 weeks of treatment. Thus, it is possible that improvement in insulin sensitivity in IL-1Ra ASO-treated mice was simply due to the lower body weight. To address this confounding variable we performed detailed measurements of whole-body and tissue specific insulin action using hyperinsulinemic-euglycemic clamps in body weight matched mice using the lighter control ASO treated mice and heavier IL-1Ra ASO treated mice in the cohort. Significantly, our results revealed that the improvements in whole-body insulin sensitivity were independent of changes in body weight. In addition, these improvements were due to significantly enhanced hepatic insulin sensitivity, with no improvements in muscle or adipose insulin sensitivity. Furthermore, inflammatory gene expression (TNF-α and CD11c) and triglyceride levels were also significantly and specifically reduced in the liver of IL-1Ra ASO treated mice compared with control treated HFD mice.

The balance of IL-1β and IL-1Ra is particularly important [9] and as IL-1β expression increases with obesity it induces the expression of IL-1Ra [20]. IL-1Ra and IL-1β share conserved sequences that allow them to both bind to the IL-1R1 but also have areas of low sequence and structural similarity which mediate their distinct biologic activities [34]. We measured IL-1Ra and IL-1β mRNA expression and noted no significant difference in IL-1β expression in liver or adipose tissue of mice treated with IL-1Ra ASO compared with control ASO suggesting IL-1Ra knockdown was specific and did not have off-target effects on IL-1β.

Importantly, we show that these insulin sensitizing effects are not mediated through the IL-1R1 as antisense knock-down of IL-1Ra in mice lacking the IL-1R1 still show improved insulin sensitivity. Although the receptor through which IL-1Ra exerts these effects in the liver remains to be identified, other studies have also reported that IL-1Ra has agonist effects at another receptor other than IL-1R1 [35], [36]. For example, Loscher et al (2003) show that incubation of IL-1Ra with synaptosomes from IL-1R1 KO resulted in decreased glutamate release while IL-1β had no effect, thus demonstrating that IL-1Ra can also signal through an alternate receptor other than the IL-1R1 [36]. At high concentrations, as found in obesity, IL-1Ra could have deleterious effects independent of IL-1R1 since IL-1R1 KO mice display improved insulin sensitivity after IL-1Ra knock-down.

Targeted IL-1 therapy is a major focus point of current anti-inflammatory approaches in the treatment of type 2 diabetes [37]. Therapeutics targeting IL-1 signaling are based on the selective blockade of IL-1R1 activation with either IL-1Ra or specific neutralizing antibodies to IL-1β, which result in lower blood glucose levels and improves β-cell secretory function [10], [38]. However, clinical studies have revealed that although anti-inflammatory treatment of type 2 diabetic patients with recombinant IL-1Ra (Anakinra) produced modest reductions in glycated hemoglobin, no difference in insulin sensitivity was observed compared with placebo [12], [13]. We propose that this can be explained by the tissue specific effects of IL-1Ra on the development of insulin resistance.

Previous studies have demonstrated an important interplay between IL-1 signaling, energy homeostasis and body weight regulation. For example, IL-1R1 deficient mice develop mature onset obesity [39], [40] while IL-1Ra KO mice are leaner than their littermates [22], [23]. High levels of IL-1Ra may be linked to the development of obesity-induced leptin resistance, as direct injection of IL-1Ra to the cerebral ventricle can inhibit the suppressive effects of leptin on food intake and blunt leptin mediated increase in core body temperature [14], [41]. Interestingly, we found that IL-1Ra ASO treatment decreased body weight of HFD-fed, obese mice, despite similar food intake and thus is unlikely to have affected appetite. Our energy expenditure data suggest that the leaner body weight is at least in part due to higher oxygen consumption, as well as a higher CBT. IL-1Ra knock-down in HFD-fed mice resembles the phenotype previously observed in IL-1Ra KO mice, whom display increased energy expenditure, increased oxygen consumption and higher CBT [22], [23], [42]. The reasons for these effects on energy expenditure could be due, in part, to the noted up-regulation of UCP1 in BAT in IL-1Ra-ASO treated HFD mice and the pyrogenic effects of IL-1β [43].

In summary, we show that normalization of IL-1Ra improves insulin sensitivity in obese, insulin resistant mice. This mechanism is independent of the classical IL-1R1 signaling and we hypothesize that at high concentrations, as observed in chronic obesity, IL-1Ra also binds to another, yet to be identified, receptor and drives the development of hepatic insulin resistance. These findings have important consequences in current and future anti-inflammatory approaches in the treatment of Type 2 diabetes.

## Supporting Information

Figure S1 A. QPCR analysis of IL-1Ra expression after 6 wks of treatment in various tissues, B. QPCR analysis of Glucose 6-phosphatase(G6Pase) and PCK1 liver expression in IL-1a ASO and control ASO treated mice after 6 wks of treatment. C. Plasma concentration of Interleukin 10 (IL-10), Interleukin 1 beta (IL-1β), Interleukin 6 (IL-6), Monocyte chemotactic protein 1 (MCP-1) and Tumor necrosis factor alpha (TNF-α) after 6 weeks of IL-1Ra ASO or control ASO treatment.
(TIF)

Figure S2 **A–D: GTT and ITT in WT IL1Ra ASO or control ASO treated mice.** A) GTT, presented as % change from initial blood glucose (Treatment p = 0.5084, Time p<0.0001, Interaction p = 0.0103), B) Area under the curve for GTT, C) ITT presented as % change from initial blood glucose, (Treatment p = 0.0034, Time p<0.0001, Interaction, p<0.0001) D) Area under the curve for ITT. E–H, GTT and ITT in IL-1R1 KO mice treated with IL-1Ra or control ASO E) GTT, presented as % change from initial blood glucose, (Treatment p = 0.274, Time p<0.0001, Interaction, p = 0.0219. F) Area under the curve for GTT, G) ITT, presented as % change from initial blood glucose ASO (Treatment p = 0.2169, Time p<0.0001, Interaction, p = 0.1212. H) Area under the curve for ITT.
(TIF)

Table S1 **Primer information.**
(TIF)
