# Supplementary figures and images for: Knock-Down of IL-1Ra in Obese Mice Decreases Liver Inflammation and Improves Insulin Sensitivity
Source: PLoS One. 2014 Sep 22;9(9):e107487. doi: 10.1371/journal.pone.0107487 (PMC4171490; doi:10.1371/journal.pone.0107487)

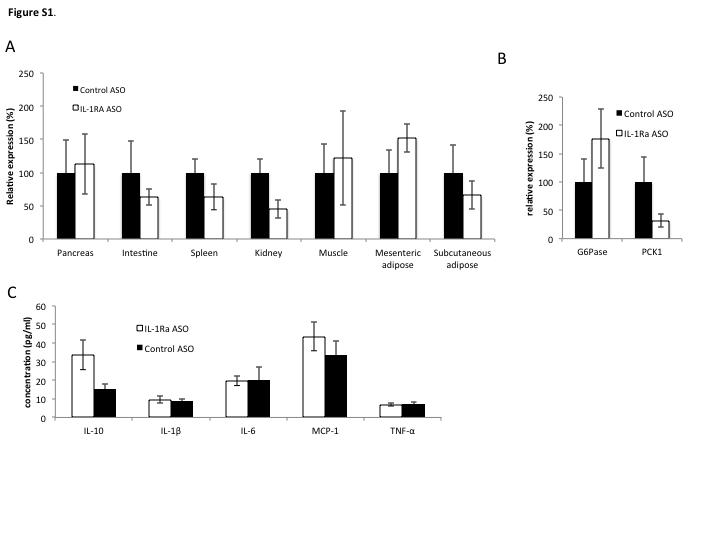

Supplement: Figure S1 — A. QPCR analysis of IL-1Ra expression after 6 wks of treatment in various tissues, B. QPCR analysis of Glucose 6-phosphatase(G6Pase) and PCK1 liver expression in IL-1a ASO and control ASO treated mice after 6 wks of treatment. C. Plasma concentration of Interleukin 10 (IL-10), Interleukin 1 beta (IL-1β), Interleukin 6 (IL-6), Monocyte chemotactic protein 1 (MCP-1) and Tumor necrosis factor alpha (TNF-α) after 6 weeks of IL-1Ra ASO or control ASO treatment. (TIF) [file pone.0107487.s001.tif]

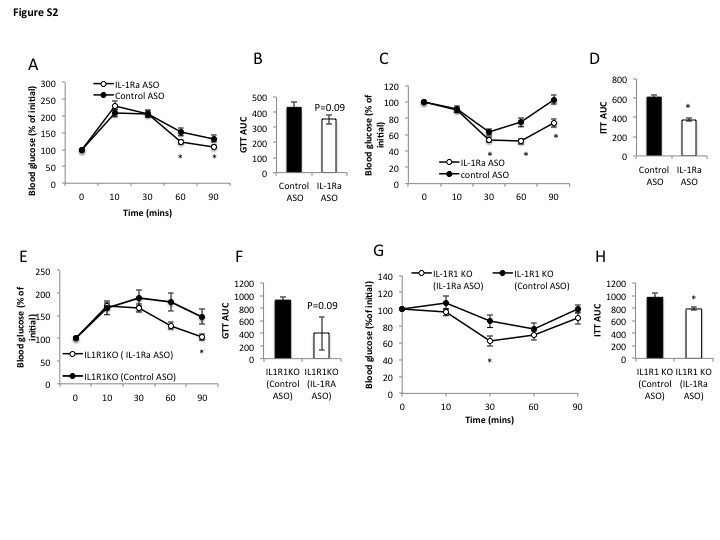

Supplement: Figure S2 — A–D: GTT and ITT in WT IL1Ra ASO or control ASO treated mice. A) GTT, presented as % change from initial blood glucose (Treatment p = 0.5084, Time p<0.0001, Interaction p = 0.0103), B) Area under the curve for GTT, C) ITT presented as % change from initial blood glucose, (Treatment p = 0.0034, Time p<0.0001, Interaction, p<0.0001) D) Area under the curve for ITT. E–H, GTT and ITT in IL-1R1 KO mice treated with IL-1Ra or control ASO E) GTT, presented as % change from initial blood glucose, (Treatment p = 0.274, Time p<0.0001, Interaction, p = 0.0219. F) Area under the curve for GTT, G) ITT, presented as % change from initial blood glucose ASO (Treatment p = 0.2169, Time p<0.0001, Interaction, p = 0.1212. H) Area under the curve for ITT. (TIF) [file pone.0107487.s002.tif]

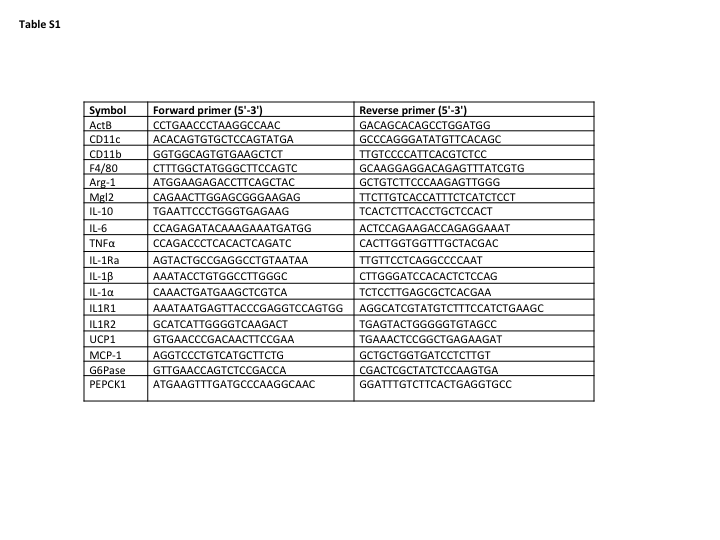

Supplement: Table S1 — Primer information. (TIF) [file pone.0107487.s003.tif]
